# Association between migration and physical activity among medical students from a university located in Lima, Peru

**DOI:** 10.1371/journal.pone.0212009

**Published:** 2019-02-27

**Authors:** Alejandro Zevallos-Morales, Leslie Luna-Porta, Henry Medina-Salazar, María Yauri, Alvaro Taype-Rondan

**Affiliations:** 1 Universidad de San Martín de Porres, Facultad de Medicina Humana, Lima, Peru; 2 Universidad San Ignacio de Loyola, Unidad de Investigación para la Generación y Síntesis de Evidencias en Salud, Lima, Peru; University of Mississippi Medical Center, UNITED STATES

## Abstract

**Objective:**

To evaluate the association between migration and physical activity among medical students from a university located in Lima, Peru.

**Methods:**

A cross-sectional study was conducted among second-year medical students from a Peruvian university. Data on moderate- to vigorous-intensity physical activity (MVPA) and migration features were obtained through a self-report questionnaire. To assess the associations of interest, prevalence ratios (PR) along with their 95% confidence intervals (95% CI) were calculated using Poisson regression with robust variances.

**Results:**

We analyzed data from 312 students (54.5% were women, mean age: 19.0 years, standard deviation: 1.4 years), 90 (28.9%) students performed MVPA for ≥150 minutes/week, 118 (37.8%) performed MVPA for ≤30 minutes/week, and 114 (36.7%) were migrants. Being a migrant was not associated with performing MVPA for ≤30 nor ≥150 minutes/week. However, adjusted analysis showed that the frequency of performing MVPA for ≤30 minutes/week was greater among those who migrated less than five years ago (PR: 1.43; 95% CI: 1.05–1.93) and among those who migrated to continue their studies (PR: 1.44; 95% CI: 1.06–1.94), compared to non-migrants.

**Conclusion:**

In our population, being a migrant was not associated with physical activity. However, low physical activity was more prevalent among recent migrants and among those who had migrated to study, compared to non-migrants.

## Introduction

Physical inactivity is associated with an increased risk of developing chronic diseases such as type 2 diabetes mellitus, hypertension, and some types of cancer. [1] Also, physical inactivity is considered as the fourth leading risk factor for mortality worldwide, being responsible for 5.3 million premature deaths in 2008. [2, 3] Nonetheless, it is estimated that 31.1% of adults worldwide are physically inactive (defined as not engaging in 30 minutes of moderate-intensity physical activity on at least 5 days every week, not engaging in 20 min of vigorous-intensity physical activity on at least 3 days every week, and not achieving a total of 600 metabolic equivalent-min per week). [4]

Physical activity can be measured using different strategies: Self-report measures are the most widely used, and much of worldwide data on physical activity are obtained through these measures. On the other hand, direct measures (including accelerometers, pedometers, heart-rate monitors, and multiple-sensor devices) are being used in a growing number of studies. [5]

Previous studies have found lower rates of physical activity in more urbanized areas compared to less urbanized areas, [6] and that those who migrate from less urbanized towards more urbanized locations tend to decrease their physical activity. [1, 6–11] Considering that migration usually occurs towards more urbanized areas and that around 3 million people migrate to urban cities weekly worldwide, [12] the association between migration and physical activity constitutes a public health issue.

In Latin America, many of the migrants are young, and they choose or are forced to migrate seeking better economic, academic or job opportunities. [13, 14] Since it has been observed that physical activity patterns in youth are an important predictor of physical activity in adulthood, [15] the potential decrease of physical activity in young migrants might have a great impact on this population health.

Currently, there is a paucity of information regarding the association between migration and physical activity in young people, making it difficult to develop and implement adequate preventive interventions. Moreover, young people who migrate to pursue time-consuming careers such as medicine may have less leisure time and, therefore, perform less physical activity. Thus, the objective of this study was to evaluate the association between migration and physical activity among medical students from a university located in Lima, Peru.

## Methods

### Study design

A cross-sectional study was conducted during May 2016 among second-year medical students from Universidad de San Martín de Porres (USMP) located in Lima, the capital city of Peru.

At USMP, undergraduate medical education lasts 7 years, which are divided into 3 groups: basic studies (first, second, and third years), clinical studies (fourth, fifth, and sixth years) and internship (seventh year).

Lima, the capital city of Peru, has over 9 million inhabitants as of 2018.[16] Internal migration in Peru is fairly common and Lima is the primary destination. Between 2002 and 2007, approximately 1.5 million people migrated to another location within Peru, of which 42.8% migrated to Lima. [17]

### Participants and procedures

The population consisted of second-year medical students enrolled in the Biostatistics course (one of the obligatory courses of the second-year of the career). Four hundred and sixteen students were enrolled in this course during the first semester of 2016. We included all students who accepted to participate in this study and excluded those who were less than 18 years old and those who did not complete the variables of interest.

Biostatistics students were distributed among 30 classes. The distribution of students per class was granted by the Department of Basic Studies of USMP. Subsequently, four previously trained interviewers attended these classes to coordinate with the professors, perform informed consent to the students, and administer the questionnaires to all those who accepted to participate. This was conducted at the beginning or at the end of the class, according to availability. Students were given enough time to complete the questionnaires. Later, two authors entered the data from questionnaires in parallel into a Microsoft Excel database, and a third author reviewed for inconsistencies.

### Outcome: Physical activity

To assess self-reported physical activity, we asked participants: “*Please*, *calculate the number of minutes per week you engage in moderate- to vigorous- intensity physical activity (MVPA) during a regular week of classes*.” In this case, MVPA was defined as any activity that involves physical wear and heart rate increase, such as brisk walking, jogging, bike riding, swimming, practicing a sport, among others, following the definition proposed by the Canadian Society for Exercise Physiology (CSEP). [18]

The CSEP recommends that adults aged 18–64 years should engage in at least 150 minutes of MVPA weekly. [18] Accordingly, we defined adequate physical activity as performing MVPA for ≥150 minutes/week. In addition, we defined low physical activity as being in the lowest tertile of MVPA (≤30 minutes/week).

### Exposure: Migration and characteristics

The exposures of interest were migration features (migration status, degree of urbanization of the place of origin, years lived in Lima, and reason for migration). Other variables evaluated were age and sex.

To define migration status, we used the question: “*Have you lived anywhere other than Lima*?” Those who answered yes were considered migrants, and those who answered no were considered non-migrants.

To evaluate the degree of urbanization of the place of origin of the participants, we asked where they were born and where they lived before moving to Lima. Then, we divided migrants into two groups: those who came from a big city (if they were born in a big city or had lived in a big city before migrating to Lima), and those who came from a small city (if they were born in a small city and had lived in a small city before migrating to Lima). We used the median of the population of the places of origin of our migrants (500,000 people) as the cutoff point to categorize cities in big and small. The population number was estimated using national and international population data accordingly.

To know the number of years lived in Lima, we used the question: “*How many years have you been living in Lima*?” Then, we divided migrants into two groups: late migrants (if they had migrated five or more years ago), and recent migrants (if they had migrated less than five years ago). This was an arbitrary cut-off point decided by the authors.

To know the reason for migration, we used the question: “*Why did you move to Lima*?*”* Then, we divided migrants into two groups: migrated to continue their studies and migrated for other reasons (such as work, health, or because his/her family migrated).

### Ethics

Ethical approval was obtained from the Institutional Review Board at Hospital Nacional Docente Madre-niño "San Bartolomé" (RCEI-40). In addition, we obtained the signed consent from the participants before applying the questionnaires.

### Statistical analysis

We used STATA v.14 for the analysis. We did a descriptive analysis of the population using absolute and relative frequencies as well as mean ± standard deviations. Then, we assessed the factors associated with the two outcomes (performing MVPA for ≥150 minutes/week and for ≤30 minutes/week) by calculating crude and adjusted prevalence ratios (PR) and their 95% confidence intervals (95% CI) using Poisson regression with robust variants, as proposed by Coutinho et al. [19] Regressions were adjusted by age and sex. Lastly, we assessed the association between the years lived in Lima and the reasons for migrating using the chi-2 test.

## Results

A total of 416 second-year medical students were enrolled in the Biostatistics course, of which 356 completed the questionnaire. We excluded 44 participants (19 male and 25 female): 39 for being less than 18 years old, and 5 for not answering the physical activity question. Thus, we analyzed data from 312 students (75.0% of the total number of students).

Among those, 170 (54.5%) were women and the mean age was 19.0 ± 1.4 years. Regarding physical activity, 118 (37.8%) performed MVPA for ≤30 minutes/week and 90 (28.9%) performed MVPA for ≥150 minutes/week. With respect to migration features, 114 (36.5%) were migrants; 57 (50.0%) of them came from big cities before migrating to Lima, 67 (46.5%) had migrated less than five years ago, and 77 (53.5%) had migrated to continue their studies (Table 1).

**Table 1:** Characteristics of the population.

Characteristics	N (%)
**Sex (n = 312)**	
Male	142 (45.5)
Female	170 (54.5)
Age in years (n = 312)*	19.0 ± 1.4
**Moderate- to vigorous-physical activity (n = 312)**	
0 to 30 minutes/week	118 (37.8)
31 to 149 minutes/week	104 (33.3)
150 minutes/week or more	90 (28.9)
**Migrant (n = 312)**	
No	198 (63.5)
Yes	114 (36.5)
**Place of residence before moving to Lima (n = 308)**	
Non-migrants	198 (64.3)
Migrants who came from a big city	57 (18.5)
Migrants who came from a small city	53 (17.2)
**Years lived in Lima (n = 297)**	
Non-migrants	183 (61.6)
Late migrants (≥5 years)	47 (15.8)
Recent migrants: (<5 years)	67 (22.6)
**Reason for migration (n = 311)**	
Non-migrants	197 (63.3)
Migrated to continue their studies	77 (24.8)
Migrated for other reasons	37 (11.9)

* Mean ± Standard deviation

In the analysis adjusted by age and sex, we did not find any factor associated with performing MVPA for ≥150 minutes/week. However, students who migrated less than five years ago had a 43% higher prevalence of performing MVPA for ≤30 minutes/week compared to non-migrants, and those who migrated in order to continue their studies had a 44% higher prevalence of performing MVPA for ≤30 minutes/week compared to non-migrants. (Table 2).

**Table 2:** Factors associated with performing ≥150min and ≤30min moderate- to vigorous- intensity physical activity.

Variables	Moderate- to vigorous- intensity physical activity for ≥150min				Moderate- to vigorous- intensity physical activity for ≤30min			
	No	Yes	Crude PR (95% CI)	Adjusted * PR (95% CI)	No	Yes	Crude PR (95% CI)	Adjusted PR (95% CI)
**Sex**								
Male	94 (66.2)	48 (33.8)	Ref	Ref	95 (66.9)	47 (33.1)	Ref	Ref
Female	128 (75.3)	42 (24.7)	0.73 (0.52–1.04)	0.75 (0.53–1.06)	99 (58.2)	71 (41.8)	1.26 (0.94–1.69)	1.25 (0.93–1.68)
**Age in years**	19.0 ± 1.2	19.3 ± 1.8	**1.10 (1.00–1.20)**	1.09 (1.00–1.19)	19.1 ± 1.5	18.9 ± 1.2	0.93 (0.83–1.05)	0.94 (0.83–1.05)
**Migrant**								
No	140 (70.7)	58 (29.3)	Ref	Ref	130 (65.7)	68 (34.3)	Ref	Ref
Yes	82 (71.9)	32 (28.1)	0.96 (0.66–1.38)	0.94 (0.65–1.35)	64 (56.1)	50 (43.9)	1.28 (0.96–1.70)	1.31 (0.99–1.74)
**Place of residence before moving to Lima**								
Non-migrants	140 (70.7)	58 (29.3)	Ref	Ref	130 (65.7)	68 (34.3)	Ref	Ref
Migrants who came from a big city	40 (70.2)	17 (29.8)	1.02 (0.65–1.60)	1.00 (0.64–1.56)	32 (56.1)	25 (43.9)	1.28 (0.90–1.82)	1.30 (0.92–1.84)
Migrants who came from a small city	41 (77.4)	12 (22.6)	0.77 (0.45–1.33)	0.77 (0.45–1.34)	29 (54.7)	24 (45.3)	1.32 (0.93–1.88)	1.34 (0.94–1.92)
**Years lived in Lima**								
Non-migrants	133 (72.7)	50 (27.3)	Ref	Ref	118 (64.5)	65 (35.5)	Ref	Ref
Late migrants (≥5 years)	33 (70.2)	14 (29.8)	1.09 (0.66–1.80)	1.03 (0.62–1.72)	31 (66.0)	16 (34.0)	0.96 (0.61–1.49)	1.02 (0.65–1.59)
Recent migrants: (<5 years)	49 (73.1)	18 (26.9)	0.98 (0.62–1.56)	1.00 (0.63–1.57)	33 (49.3)	34 (50.7)	**1.43 (1.05–1.94)**	**1.43 (1.05–1.93)**
**Reason for migration**								
Non-migrants	140 (71.1)	57 (28.9)	Ref	Ref	129 (65.5)	68 (34.5)	Ref	Ref
Migrated to continue their studies	56 (72.7)	21 (27.3)	0.94 (0.62–1.44)	0.92 (0.60–1.42)	40 (51.9)	37 (48.1)	1.39 (1.03–1.88)	**1.44 (1.06–1.94)**
Migrated for other reasons	26 (70.3)	11 (29.7)	1.03 (0.60–1.77)	1.01 (0.59–1.72)	24 (64.9)	13 (35.1)	1.02 (0.63–1.64)	1.04 (0.65–1.67)

*Adjusted by age and sex

Bold text indicates a statistically significant difference

Lastly, we evaluated the association between years lived in Lima and the reasons for migrating to Lima and found that 88.1% of recent migrants and 38.3% of late migrants had migrated to continue their studies (chi-2 p-value <0.001).

## Discussion

### Main results

We analyzed self-reported information from 312 medical students, finding that a little less than a third performed MVPA for ≥150 minutes/week and a little more than a third performed MVPA for ≤30 minutes/week. Migration status was not associated with physical activity. Also, we did not find any association between migration features and performing MVPA for ≥150 minutes/week. However, recent migrants and those who migrated to continue their studies had a higher prevalence of performing MVPA for ≤30 minutes/week compared to non-migrants.

### Physical activity

Despite the CSEP recommendation for adults to accumulate at least 150 minutes/week of MVPA, only 28.9% of students met this recommendation. This rate seems to be lower than previous physical activity reports among medical students. In a Canadian university, 64% of fourth-year medical students met the CSEP recommendation. [20] In addition, 61% of students from a university in the United States and 32.3% of students from a university in India met the Center for Disease Control and Prevention (CDC) recommendation of 150 minutes of moderate physical activity or 60 minutes of vigorous physical activity weekly. [21, 22]

Previous studies have reported barriers to perform physical activity among university students, which might also be present in our population. These include high stress levels, high academic demand [23–25], lack of time [26–29], lack of knowledge of international recommendations about physical activity [30], lack of personal or family motivation [31, 32], and lack of opportunities or facilities at universities. [33] Thus, the implementation of certain university-based interventions could modify these barriers, such as promoting cycling or walking as a means of transportation, creating environments that encourage physical activity during leisure time, or offering free workshops that can work around students’ schedules, could help overcome these barriers. [34–36]

### Physical activity and migration

We did not find any association between migration status and physical activity. To our knowledge, this was the first study to evaluate this association among university students, although previous studies in the general population found that adult migrants tend to decrease their physical activity. [37–40] The lack of association in our study is probably due to the undifferentiated inclusion of migrants who came from places with a different degree of urbanization and had different length of migration.

We did not find any factor associated with performing MVPA for ≥150 minutes/week. However, the number of years lived in Lima and the reason for migrating was associated with performing MVPA for ≤30 minutes/week. This could mean that the cut-off point of 150 minutes may not be ideal for identifying the effects of migration features on physical activity. In consequence, further studies should consider the use of more than one cut-off point to evaluate factors associated with physical activity.

Compared to non-migrants, recent migrants performed less physical activity. Concomitantly, other studies in the general population have also found that recent migrants perform less physical activity. A study conducted in Canada assessed over 170,000 individuals from 2000 to 2003. They found that recent migrants and immigrants performed less physical activity compared to non-migrants. In this study, they defined recent migrant as being a resident for less than 10 years. [40] Another study conducted in Australia found that migrants performed less physical activity compared to non-migrants. This was higher in older populations, females and those who came from non-English speaking countries. [41]

These results may indicate that recent migrants are not yet well suited to the new environment (they are not sufficiently acculturated), [42] having higher levels of stress and just a few friends, which could be preventing them from doing physical activity. [43] Accordingly, a systematic review indicates that promoting the process of acculturation among migrants would be beneficial for physical activity interventions in prevention programs. [44] Thus, the implementation of activities targeted to individuals with low acculturation levels should be explored at universities. In addition, it cannot be guaranteed that recent migrants will be able to establish an exercise routine in the future, then longitudinal studies would be needed.

Those who migrated to continue their studies performed fewer physical activities compared to non-migrants. We have not found other studies that have assessed this association. The reason for this association is uncertain, possible explanations are that those who migrated to continue their studies spend more time studying or have migrated without their family, having to household tasks themselves; which decreases their time to do physical activity. Also, these migrants could not have the economic resources aimed at activities unrelated to their studies, thus leaving aside physical activity. Another explanation is that this is a fictitious association confounded by the length of migration since most of those who migrated to continue their studies are also recent migrants.

### Limitations and strengths

Our study assessed self-reported physical activity, which is a widely used method to evaluate physical activity patterns. [20, 34, 40, 43, 45] However, it should be taken into account that assessing self-reported physical activity may lead to over-or underestimations of the real physical activity.[46, 47] Furthermore, the use of a tool that was not previously validated may affect the study reproducibility, but as we mentioned earlier, we wanted to explore directly measured MVPA.

In addition, it is important to note that our participants were medical students from a single university, thus, generalization to other universities and careers would not be adequate, especially because USMP is a private university, which could have a population with higher socioeconomic status than that of public Peruvian universities. Lastly, we used the number of inhabitants in the cities as a proxy to measure the degree of urbanization. This evaluation may not be sufficiently accurate, we are not considering certain aspects of the place of origin, such as the type of terrain or elevation.

However, this is to our knowledge the first study to assess the association between migration/migration features and physical activity in young people, which are a large group of migrants worldwide. This evaluation provides a relevant understanding of this subject and opens new research subjects in this field.

## Conclusions

We assessed second-year medical students from a Peruvian university and found no association between migration status and physical activity. However, low physical activity was more prevalent in those who were recent migrants, and in those who had migrated to continue their studies, compared to non-migrants.

## Supporting information

S1 File Minimal data set: Database plos.dta. (DTA)
